# Advanced Photogrammetry to Assess Lichen Colonization in the Hyper-Arid Namib Desert

**DOI:** 10.3389/fmicb.2017.02083

**Published:** 2017-10-27

**Authors:** Graham Hinchliffe, Barbara Bollard-Breen, Don A. Cowan, Ashray Doshi, Len N. Gillman, Gillian Maggs-Kolling, Asuncion de Los Rios, Stephen B. Pointing

**Affiliations:** ^1^Institute for Applied Ecology New Zealand, School of Science, Auckland University of Technology, Auckland, New Zealand; ^2^The Genomics Research Institute, University of Pretoria, Pretoria, South Africa; ^3^Gobabeb Research and Training Centre, Gobabeb, Namibia; ^4^Departamento de Biogeoquímica y Ecología Microbiana, Museo Nacional de Ciencias Naturales, Madrid, Spain; ^5^Division of Science, Yale-NUS College, National University of Singapore, Singapore, Singapore

**Keywords:** astrobiology, computer vision, desert, GIS, lichen, microbial ecology, Namib Desert, photogrammetry

## Abstract

The hyper-arid central region of the Namib Desert is characterized by quartz desert pavement terrain that is devoid of vascular plant covers. In this extreme habitat the only discernible surface covers are epilithic lichens that colonize exposed surfaces of quartz rocks. These lichens are highly susceptible to disturbance and so field surveys have been limited due to concerns about disturbing this unusual desert feature. Here we present findings that illustrate how non-destructive surveys based upon advanced photogrammetry techniques can yield meaningful and novel scientific data on these lichens. We combined ‘structure from motion analysis,’ computer vision and GIS to create 3-dimensional point clouds from two-dimensional imagery. The data were robust in its application to estimating absolute lichen cover. An orange *Stellarangia* spp. assemblage had coverage of 22.8% of available substrate, whilst for a black *Xanthoparmelia* spp. assemblage coverage was markedly lower at 0.6% of available substrate. Hyperspectral signatures for both lichens were distinct in the near-infra red range indicating that *Xanthoparmelia* spp. was likely under relatively more moisture stress than *Stellarangia* spp. at the time of sampling, and we postulate that albedo effects may have contributed to this in the black lichen. Further transformation of the data revealed a colonization preference for west-facing quartz surfaces and this coincides with prevailing winds for marine fog that is the major source of moisture in this system. Furthermore, a three-dimensional ‘fly through’ of the lichen habitat was created to illustrate how the application of computer vision in microbiology has further potential as a research and education tool. We discuss how advanced photogrammetry could be applied in astrobiology using autonomous rovers to add quantitative ecological data for visible surface colonization on the surface of Mars.

## Introduction

Deserts are the largest terrestrial biome (Thomas, 2011) and are characterized by prolonged moisture stress and also significant UV and thermal stress (UNEP, 1992; Peel and Finlayson, 2007). These factors limit animal and vascular plant occurrence and in the most extreme desert landscapes they may be virtually absent, with microbial communities forming the dominant biological cover (Pointing and Belnap, 2012). In semi-arid and arid regions biological soil crusts are common and these comprise a complex association of bacteria, fungi, algae, lichen, and mosses (Belnap et al., 2003). Their diversity and ecological role have been comprehensively studied (Gundlapally and Garcia-Pichel, 2006; Elbert et al., 2012; Rajeev et al., 2013; Colesie et al., 2014; Rutherford et al., 2017) and they are critical to desert ecosystem stability (Pointing and Belnap, 2014). In the most extreme hyper-arid deserts more severe environmental conditions limit the occurrence of biological soil crusts and refuge communities account for much of the standing biomass. Colonization includes hypolithic cyanobacterial communities beneath quartz rocks (Warren-Rhodes et al., 2006, 2007; Pointing et al., 2007; Stomeo et al., 2013) as well as cryptoendolithic cyanobacterial and lichen communities within porous rocks (Lee et al., 2016; Archer et al., 2017) and mineral substrates (Wierzchos et al., 2006, 2012). A high degree of niche filtering between such communities has been observed, reflecting the strong selective forces involved in shaping these communities (Lee et al., 2016). Open soil in desert systems is relatively depauperate and largely supports low-biomass bacterial communities (Makhalanyane et al., 2015).

In the most extreme hyper-arid regions of the central Namib Desert additional surface biological cover comprises epilithic lichens that colonize exposed rocky surfaces (Nash et al., 1977; Lange and Green, 2008; Wirth, 2010). Lichen associations colonize the exposed quartz surfaces of desert pavement and strikingly lichens appear to thrive where no other surface colonization occurs. Whilst subsurface refuge communities rely on a mineral layer for protection from environmental extremes (Pointing and Belnap, 2012), the epilithic lichens are fully exposed and so have developed extraordinary desiccation tolerance (Kranner et al., 2008) and UV protective pigmentation strategies (Wierzchos et al., 2015). Namib Desert lichens are thought to derive their moisture from coastal fog and this is assumed to influence their colonization such that they appear visually to preferentially colonize elevated ground and exposed rock surfaces (Lange and Green, 2008).

The extreme and isolated environment of the Namib Desert makes ecological surveys challenging, and traditional ground-based estimates of lichen cover are limited in their application to meaningful landscape scales. In addition, the fragile nature and slow recovery rate for desert landscapes makes disturbance during scientific surveys a serious concern (Belnap and Eldridge, 2003; Kuske et al., 2012). For this reason, there is growing interest in field techniques that minimize environmental harm such as photogrammetry and remote sensing. These techniques are also a key component of remote sensing for other planetary surfaces in the field of astrobiology, the science of identifying whether life exists or has existed on other planets (Grady, 2001). A major focus for astrobiology has been our closest planetary neighbor Mars, and extreme deserts on Earth such as the Namib Desert are geologically and climatically the closest analogs available to Mars’ current surface environment (Fairén et al., 2010). The future search for extant microbial colonization on Mars’ surface will benefit from multiple approaches, and advanced photogrammetry techniques proven in desert analog environments on Earth may prove a powerful tool in this regard.

Here we report results from a field photogrammetry survey of Namib Desert lichens using RGB and hyper-spectral cameras. We report findings that demonstrate how advanced photogrammetry applied to standard photographic imagery can be used to accurately map colonization, discriminate between major taxa, create 3D renderings that inform colonization preference and azimuth, and be combined with hyper-spectral imagery to infer lichen health.

## Materials and Methods

### Field Location and Manual Sampling

The sampling location (S 23° 03.383′, E14° 38.071′; 105 m above sea level) was typical of the central Namib Desert terrain comprising desert pavement with quartz rocks embedded in a mineral soil substrate. This area is internationally renowned for its ‘lichen fields’, large areas where exposed quartz is colonized largely by orange and black lichens to form a spectacular and unusual microbial landscape. The field survey was made in April 2016 during an expedition hosted by the Gobabeb Research and Training Centre^1^. Lichen colonization comprised either orange or black-pigmented lichen thalli. Both types of lichen thalli comprised multiple species of a single genus co-existing on the quartz substrate. The orange lichens all belonged to *Stellarangia* spp. and the black lichens were *Xanthoparmelia* spp. (Wirth, 2010; Arup et al., 2013).

### Equipment and Photography

The camera used for the terrestrial photography was a Nikon D7200 24.2MP DSLR, a high-end prosumer model, combined with an 18–200 mm f/3.5–5.6 lens. The photographs for this survey were taken in bright afternoon direct sun with focal lengths between 48 and 50 mm (equivalent to 72–75 mm on a 35 mm camera). Images were captured using ISO 100 and f/8 to ensure minimal image noise and a wide depth of field. In total, 95 images were acquired in 3 passes, each a circle around the target mound at differing height and angle to ensure a high level of photographic overlap. Hyperspectral measurements were acquired using an ASD Handheld2 VNIR Spectroradiometer. This instrument allowed for readings across 325–1075 nm covering the visible and near-infrared portion of the spectrum. This was employed to measure the reflectance spectrum of light from lichens for a given pixel, as well as background substrate measurements. Wavelengths included the UV and near-UV wavelengths typically associated with photo-protective pigments, visible light including photosynthetically active wavelengths, as well as near-infra red range associated with water molecules. Calibration measurements were obtained against a Spectrolon reference disk prior to each sampling.

### Structure from Motion Photogrammetry

Structure from Motion (SfM) is a process for obtaining 3D information from a series of 2D images. It is seen as a revolution in the field of geospatial science, as compared to traditional photogrammetry techniques which require detailed *a priori* camera position. The SfM approach solves the geometric location of features in a scene and those of camera positions and orientations simultaneously (Westoby et al., 2012).

Recent developments in the field computer vision have resulted in the release of a number of commercial and open-source software packages. This project utilized RealityCapture^2^ for the photogrammetric reconstruction of the Lichen field, including the initial camera alignment and sparse point cloud generation followed by densification and 3D meshing. The resulting model comprised 78.5 million triangles and 39.4 million points, exported as a colourised point cloud for further analysis.

### Geospatial Analysis

Point data over the site were analyzed using an open source point cloud visualization software CloudCompare^3^ for the discrimination of lichen species based on color. Classification raster and Digital Surface Model (DSM) raster datasets were further exported to ESRI ArcGIS software for additional processing including slope and aspect analysis.

## Results and Discussion

Visual characterization of microbial habitats and colonization using advanced photogrammetry is an emerging and potentially powerful tool in microbial ecology. This approach has particular relevance in fragile or poorly accessible habitats such as deserts and has several advantages over conventional field sampling surveys that involve physical displacement or removal of samples, since recovery of disturbed desert microbial communities may occur on decadal or even longer timescales (Belnap and Eldridge, 2003). Furthermore, the technique may have important applications in the search for extant life on the surface of Mars (Fairén et al., 2010). Here we report a proof of concept study that demonstrates how advanced photogrammetry can be applied to accurately determine colonization and identity of desert lichens without disturbance.

**FIGURE 1:**
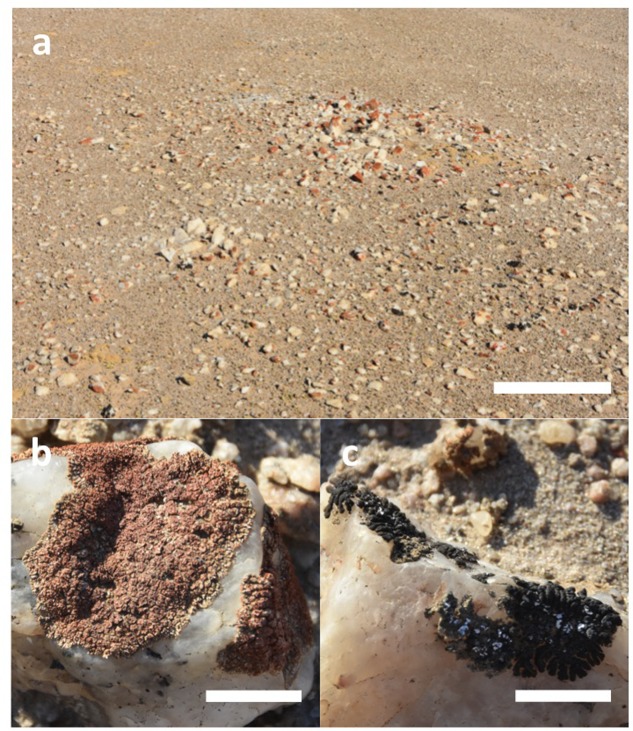
**(a)** Elevated mound supporting epilithic lichens on quartz pavement in the Namib Desert (scale bar 1 m). **(b)** Orange lichen *Stellarangia* spp. (scale bar 10 mm). **(c)** Black lichen *Xanthoparmelia* spp. (scale bar 10 mm). Each genus was encountered as multiple morphospecies co-existing within the same quartz fragments.

**FIGURE 2:**
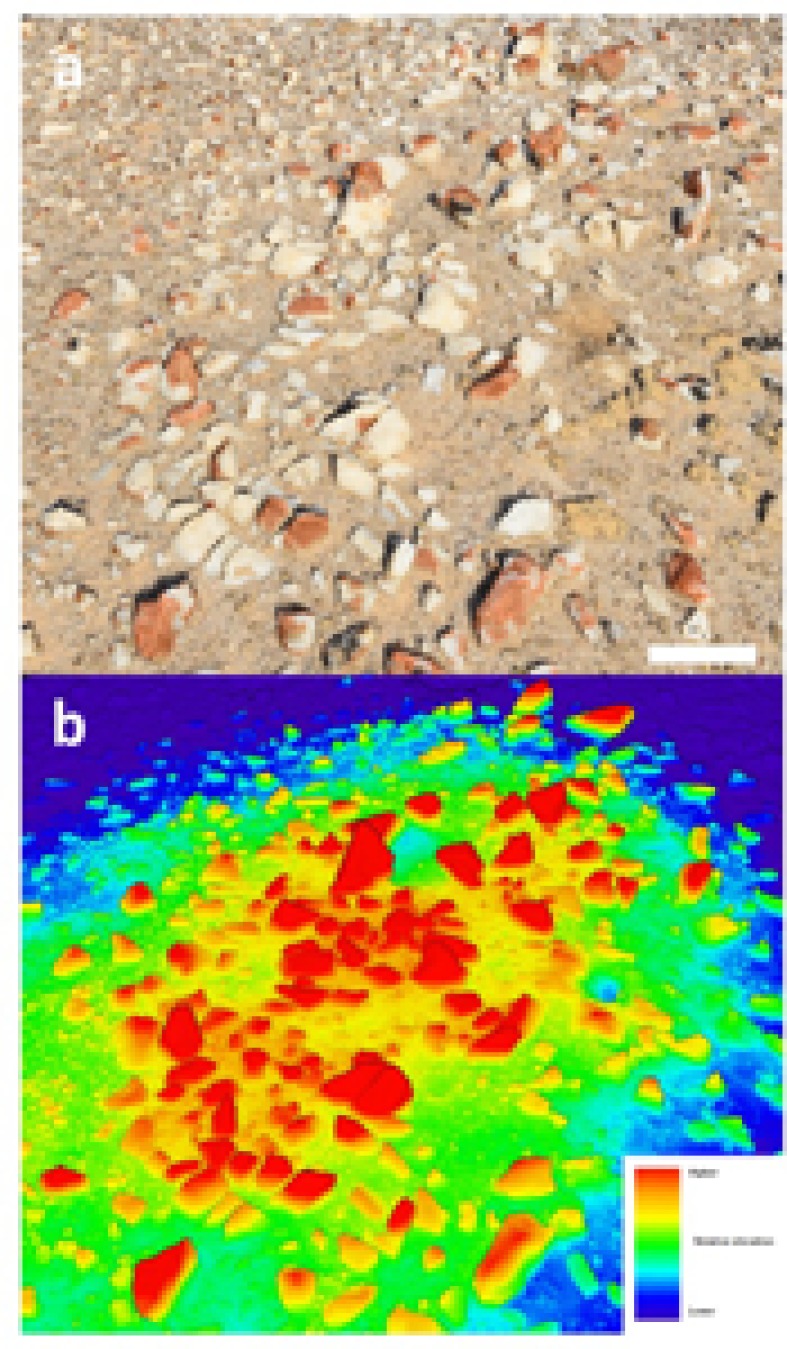
**(a)** Photograph of elevated mound section showing colonized quartz (scale bar 10 cm); **(b)** heat-map based upon the 3D point cloud showing relative elevation above surface.

The quartz pavement study site was characterized by numerous elevated mounds up to several meters in diameter where colonization was more pronounced than at elevations several centimeteres lower. We focused our activity on a single mound with the intention to demonstrate the feasibility of photogrammetry for lichen surveys (**Figure 1a**). This area was colonized by orange *Stellarangia* spp. and black *Xanthoparmelia* spp. (**Figures 1b,c**). Initially lichens were classified within the 3D point cloud based on spectral properties measured using the RGB camera, the distinctive orange color of *Stellarangia* spp. was identified through a simple threshold for the ratio of red to blue and green. The point cloud was also visualized showing relative local elevation (**Figure 2**). This revealed that exposed quartz surfaces were the most elevated parts of the micro-habitat. This supports field observations that lichens colonize these surfaces in order to access moisture from coastal fog events (Lange and Green, 2008), a strategy that has also been proposed for hypolithic microbial communities beneath quartz (Warren-Rhodes et al., 2006, 2007; Azúa-Bustos et al., 2011) and those colonizing deliquescent substrates in other deserts (Davila et al., 2008; Wierzchos et al., 2015). Interestingly the exploitation of deliquescent substrates has been postulated as a potential strategy for any extant life on the surface of Mars in order to access moisture that would otherwise be biologically unavailable as well as during stochastic moisture events (Davila et al., 2010). Our findings further suggest that a consideration of local terrain is also likely to be a key predictor in the search for habitable refuges on Mars, since colonization by Namib Desert lichens clearly occurred on surface features that facilitated marginal gains in moisture availability. The quartz substrate itself may also be important, since they display a generally cooler thermal regime than surrounding soil and so may act as condensation water collectors during fog events (Lange and Green, 2008; Azúa-Bustos et al., 2011).

**FIGURE 3:**
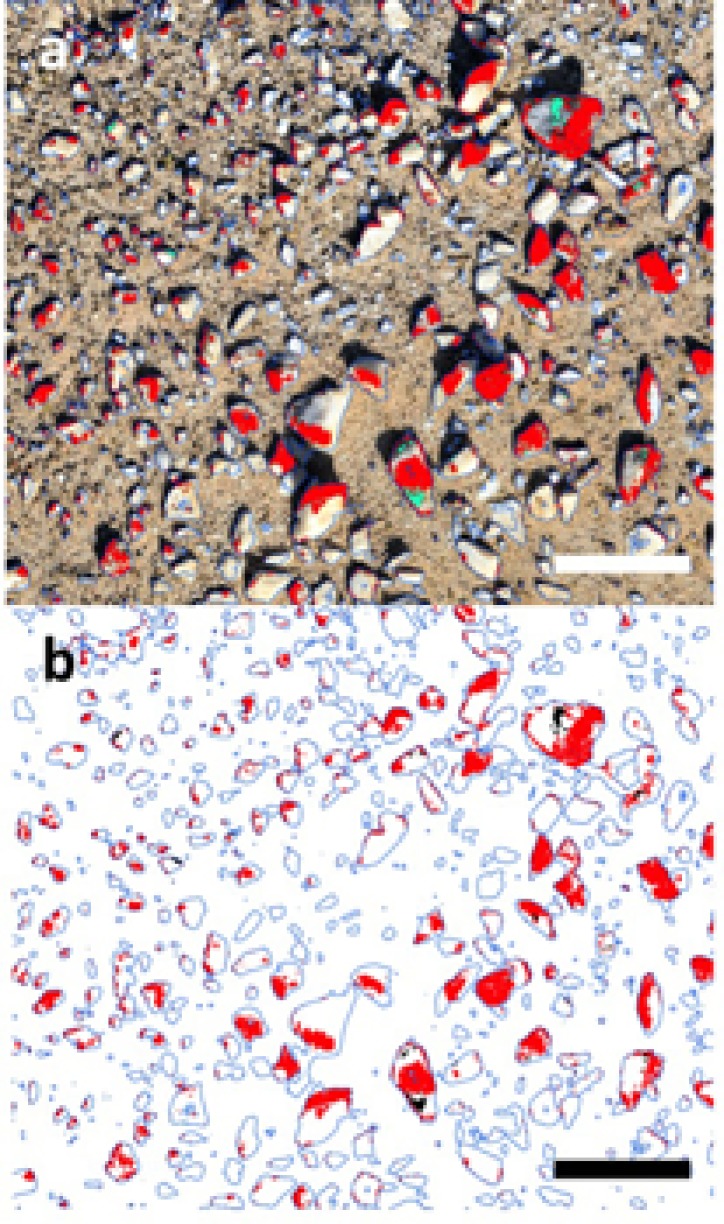
Coverage estimates for lichen based upon transformed 2D mosaic imagery using Eye Dome Lighting. **(a)** Original image, red = orange lichen, green = black lichen (scale bar 10 cm); **(b)** transformed digital image, red = orange lichen, 22.8% cover of available substrate; green = black lichen, 0.6% cover of available substrate (scale bar 10 cm).

Transforming the 3D model to a 2D orthographic projection allowed an estimate of lichen coverage on quartz surfaces (**Figure 3**). The orange *Stellarangia* spp. had coverage of 22.8% of available substrate, whilst for the black *Xanthoparmelia* spp. coverage was markedly lower at 0.6% of available substrate. This difference may reflect several unmeasured factors such as adaptation to micro-climate and habitat and/or duration of colonization. It is interesting to speculate that an albedo effect may result in hotter temperatures for the black lichen and this may be a factor limiting colonization in hot deserts where irradiance and ambient temperatures are extremely high. It is worth noting that the orthographic transformation used in the coverage estimates relies on creation of a 2-dimensional image from 3-dimensional imagery and so the ‘top down’ view that is used for this does introduce a degree of bias into coverage estimates for the 3-dimensional substrate. Further refinement of this approach will allow this bias to be better understood and addressed, although for comparative studies it is unlikely to present a major issue. This method of coverage assessment can readily be scaled up to broader spatial scales (several km^2^) using both rotary and fixed wing unmanned aerial vehicles (UAV) and we have recently applied the technique to airborne surveys of microbial mats on landscape spatial scales in terrestrial Antarctic deserts (Bollard-Breen et al., 2015). In deserts the UAV approach could also be applied to vascular plants that display characteristic patterns of colonization related to resource limitation (Rietkerk et al., 2004), since although satellite multispectral imaging has been used to map vascular plants in deserts the resolution remains limited (Adam et al., 2017).

**FIGURE 4:**
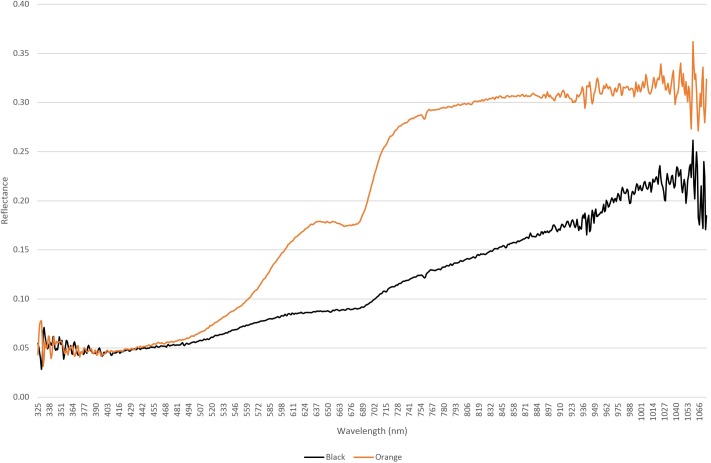
Hyperspectral reflectance curves for orange lichen *Stellarangia* spp. and black lichen *Xanthoparmelia* spp.

A further data set was captured using a hyper-spectral sensor that recorded reflectance from orange and black lichens from 325 to 1066 nm (**Figure 4**), and this mirrored observations for other microbially dominated biological covers in drylands (Weber et al., 2008; Chamizo et al., 2011). The curves for both lichens were distinct from that of the quartz substrate and showed characteristic dips in reflectance in the chlorophyll wavelengths (approximately 700 and 750 nm). The lower reflectance at near UV and UV wavelengths for both lichens likely occurred due to absorbance by melanised pigments and/or depisdones that are commonly produced by lichen mycobionts (Solhaug et al., 2003). At higher wavelengths anthraquinone-like compounds that account for the characteristic orange-yellow color of some lichens and are produced as photo-protective accessory pigments for chlorophyll typically absorb strongly at blue wavelengths (Solhaug and Gauslaa, 1996). Reflectance in the near infra-red region is associated with plant cell moisture status and can indicate plant health (Peñuelas and Filella, 1998). The near threefold difference between lichens in this range could therefore indicate that the orange lichen thalli supported a relatively more hydrated photoautotrophic biomass compared with the black lichens.

**FIGURE 5:**
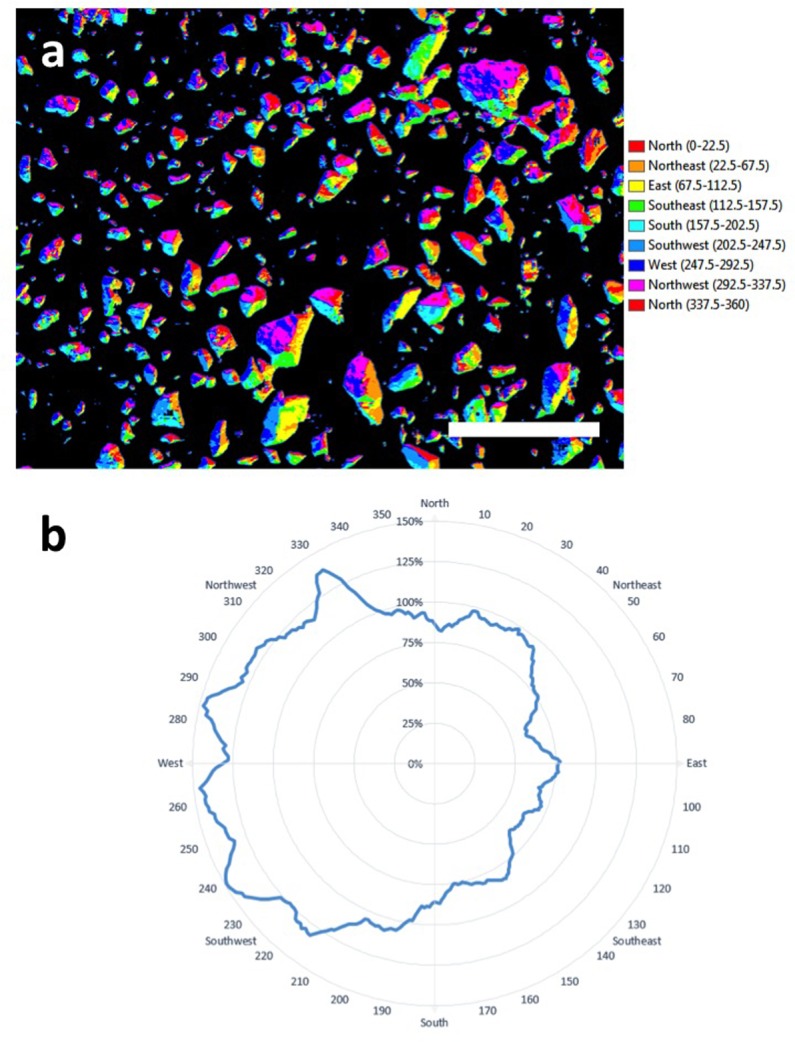
**(a)** Aspect-map showing azimuth for colonized surfaces on quartz (scale bar 10 cm); **(b)** plot of azimuth for colonization showing a clear preference by lichens for west-facing surfaces.

An important aspect of lichen ecology in extreme deserts is their source of moisture since this is the major limiting factor to survival in deserts (Pointing and Belnap, 2012). Further transformation of the data and assignment of surfaces to a given azimuth allowed creation of an aspect map showing azimuth for colonized surfaces on quartz (**Figure 5**). The polar plot showed that at the micro-habitat scale both orange and black lichens displayed a clear preference for colonization of west-facing surfaces. This is consistent with prevailing sources of coastal fog in this desert and so the data adds strong empirical support to the proposal that fog is the major driver of lichen colonization in the Namib Desert (Lange and Green, 2008). The thallus structure of lichens colonizing elevated substrates can be envisaged as effective water collectors, in contrast to refuge communities beneath rocky substrates that rely on an indirect moisture transfer mechanism from substrate to biomass (Pointing, 2016).

The computer vision techniques applied to these data have clear applications in the search for traces of life on the surface of Mars. Whilst there is a strong argument that any extant life today is likely to be chemotrophic and anaerobic (Westall et al., 2015) and thus comprise simple prokaryotic cells, the symbiotic relationship within lichens evolved as an adaptation to environmental stress and was likely ancestral to many extant free-living fungal taxa (Lutzoni et al., 2001). As such lichens are a useful model for any potential symbiotic associations on other planetary surfaces and both cryptoendolithic and epiphytic lichens have been demonstrated as capable of surviving extended periods in outer space (Sancho et al., 2007; Onofri et al., 2012) and simulated Mars-like conditions (de Vera et al., 2004; de Vera et al., 2010). The image capture data presented here could feasibly be acquired from surface rover-mounted cameras on Mars surface and used to identify biological colonization as well as important supporting ecological data such as substrate and aspect preference.

A further value for photogrammetry data in microbial ecology lies in its potential value in education and outreach. We created a 3-dimensional ‘fly through’ video (**Supplementary Video File**) to illustrate how lichen colonization occurs at the micro-habitat scale^4^ and this has clear value in allowing learners and the general public to visualize fundamentals of microbial colonization in desert research and visualize concepts in astrobiology. Ongoing refinement with user interface options such as interactive callouts and transferring 3D data to virtual reality environments will undoubtedly see the rapid emergence of new resources for many habitats and applications.

## Conclusion

We have demonstrated the feasibility of using advanced photogrammetry to accurately map lichen coverage and discriminate between lichen taxa in the central Namib Desert. The method also allows important micro-habitat related information to be determined such as the micro-topography of colonization on the quartz substrate. The imaging systems could readily be carried as payload by fixed wing and rotary UAV to provide important landscape scale low-impact ecological surveys for deserts.

## Author Contributions

SP, AdLR, and DC conceived the study. BB-B, DC, AD, LG, AdLR, and SP conducted the fieldwork. GM-K provided fieldwork logistical support and local knowledge. GH conducted computer vision and GIS analysis. AdLR conducted lichen identifications. SBP wrote the manuscript. All authors read and commented on the draft manuscript.

## Conflict of Interest Statement

The authors declare that the research was conducted in the absence of any commercial or financial relationships that could be construed as a potential conflict of interest.

## Footnotes

1 http://www.gobabebtrc.org/

2 http://www.capturingreality.com

3 http://www.cloudcompare.org/

4 https://youtu.be/aoUsJRxnV84

## Supplementary Material

**FILE |** 3D video fly-through of elevated mound showing quartz pavement colonised by orange and black lichens. Accessible online: https://youtu.be/aoUsJRxnV84
